# Human Milk Sampling Protocols: Is the Mean Macronutrient Composition of Pre- and Post-Feed Samples the Average of One Entire Feed?

**DOI:** 10.1177/08903344251389605

**Published:** 2025-11-29

**Authors:** Wietske Verveld, Johanna Rebecca de Wolf, Chris Giovanni Legtenberg, Nienke Bosschaart

**Affiliations:** 1 Biomedical Photonic Imaging Group, TechMed Centre, University of Twente, Enschede, The Netherlands

**Keywords:** breastfeeding, breastfeeding assessment, feeding patterns, human milk, human milk collection, infant nutrition, infrared spectroscopy, lactation, milk composition, observational study

## Abstract

**Background:**

Human milk sub-sampling protocols are used in lactation research to estimate milk composition, while minimizing interference with normal breastfeeding. However, macronutrient concentrations in human milk can be highly variable, and the accuracy of sub-sampling protocols for a single breastfeed is currently unknown.

**Aim:**

We investigated the accuracy of three milk sub-sampling protocols for estimating the macronutrient concentrations of a complete feed: the mean of pre- and post-feed samples, pre-feed samples only, and post-feed samples only.

**Method:**

In this observational study, macronutrient concentrations from each sub-sampling protocol were compared to the volume-weighted average of the complete pumping session, based on foremilk, bulk milk, and hindmilk samples from 15 mothers. Macronutrient concentrations of each milk fraction were measured with a human milk analyzer. Additionally, correlations between macronutrient concentrations and lactation characteristics were studied.

**Results:**

Macronutrient concentrations from each sub-sampling protocol were strongly correlated with the volume-weighted average reference concentrations. Significant biases were found for the fat concentration (between -1.3 and +1.9 g/dl, depending on the protocol), but not for protein and carbohydrate concentrations.

**Conclusions:**

For the fat concentration, none of the three sub-sampling protocols was accurate. The mean of pre- and post-feed samples could, however, be used for high-fat milk samples where measurement errors exceed the bias (+0.3 g/dl). Pre-feed or post-feed samples only should not be used to estimate the fat concentration of a complete feed. For proteins and carbohydrates, one milk sample taken at any moment in a breastfeed is sufficient to represent the concentration in the complete feed.

## Key Messages

- We assessed the accuracy of three sub-sampling protocols for estimating macronutrient concentrations in a single breastfeed.
- Pre-feed samples only, or post-feed samples only, should not be used to estimate the fat concentration of a complete feed. The mean of pre- and post-feed samples can be used for high-fat milk samples, but should not be used for low-fat milk samples, because it overestimates the fat concentration.
- For proteins and carbohydrates, one milk sample taken at any moment in a breastfeed is sufficient to represent the concentration in the complete feed.
- This study gives a detailed insight into the role of sub-sampling accuracy to support the choice of a milk sampling strategy in lactation research.

## Background

It is well established that breastfeeding brings numerous health benefits to breastfed infants and their mothers (Masi & Stewart, 2024; Victora et al., 2016). However, the relationship between infant health outcomes and the composition of human milk is still a major area of interest within the field of human lactation research (Ahuja et al., 2022; Geddes et al., 2021; Suwaydi, Lai, Gridneva, et al., 2023). The composition of human milk is complex and dynamic. It is dependent on maternal factors (e.g., gestational age, diet, genetics), infant factors (e.g., biological sex of the infant at birth), and feeding factors (e.g., biorhythm, lactation stage), but the composition of human milk also varies within a feed (Baumgartel, 2024; Hartmann et al., 1986; Hytten, 1954; Italianer et al., 2020; Saarela et al., 2005; Suwaydi, Lai, Rea, et al., 2023). Fat is the most variable macronutrient in human milk, as lipid concentrations can increase by a factor of two to five from the initial milk that is secreted during a breastfeed (foremilk), to the last fraction of secreted milk (hindmilk) (de Wolf et al., 2021; Hall, 1979; Neville et al., 1984; Veenstra et al., 2019).

Due to the highly variable nature of human milk, sampling protocols should be chosen carefully. Complete breast expression (manual or with a pump) would give the most accurate values for the expressed milk volume and composition; however, for two important reasons, this method is not always preferred. First, the expressed volume is not necessarily equal to the normal volume that an infant would consume (Drewett & Woolridge, 1981; Jackson et al., 1988). Second, it is often chosen to minimize the interference of research and diagnostics on normal breastfeeding, especially in longitudinal studies that require multiple milk samples (Leghi et al., 2020). Therefore, it is desirable to maximize the amount of milk that an infant can consume directly from the breast. Instead of analyzing complete feeds, the standard for human milk collection is sub-sampling and pooling of milk samples from each feed during a 24-hour period (Ballard & Morrow, 2013; Leghi et al., 2020; Suwaydi, Lai, Gridneva, et al., 2023). Different sub-sampling protocols exist that obtain either one small milk sample before a feed (pre-feed), during a feed (mid-feed), after a feed (post-feed), or both pre- and post-feed (George et al., 2020; Leghi et al., 2020; Suwaydi, Lai, Gridneva, et al., 2023).

To date, protocol validation for estimating infant milk intake during a day has focused on volumetric averaging protocols (Rios-Leyvraz & Yao, 2023; Scanlon et al., 2002; Suwaydi, Lai, Gridneva, et al., 2023), and different sampling moments (George et al., 2020). However, the true macronutrient intake within one feed is often estimated from the mean of pre- and post-feed macronutrient concentrations without evidence-based validation. This infant-friendly protocol was first used by Prentice et al. (1981), who observed in the data of Hytten (1954) that “a reasonable approximation of the mean feed fat concentration (average error 5%) can be obtained by taking the mean of the fat levels in the first and last milks to be drawn from the breast” (Prentice et al., 1981, p. 484).

The pre- and post-feed sampling protocol was subsequently applied by Hartmann et al. (1986), but the accuracy of this protocol was already a topic of discussion, as the milk fat concentration was known to be nonlinear over time during a feed or pumping session (Hytten, 1954; Watson et al., 1982). In 1989, Woodward et al. elaborated further on this nonlinear behavior of milk fat concentration with a model that predicted the fat concentration would increase during one feed with an exponent of 1.35. This model would give an overestimation of the fat content when the pre- and post-feed concentrations were averaged, while Hartmann et al. (1986) predicted there would be an underestimation when using their method compared to the actual fat content of one entire feed.

Leghi et al. (2020) found that the average milk fat concentration in studies using complete expression was similar to the average fat concentration from studies using pre- and post-feed sampling. However, this does not guarantee that the average of pre- and post-feed samples gives accurate concentrations for an individual milk sample, because the compared studies had different cohorts. Despite these uncertainties, the mean of the pre- and post-feed concentrations has been used as a sub-sampling protocol in many studies that evaluated macronutrient variations and infant macronutrient intake (George et al., 2020; Gridneva et al., 2018, 2019, 2022; Khan et al., 2013; Mitoulas et al., 2002; Suwaydi, Lai, Rea, et al., 2023).

The aim of this study is to clarify the uncertainty concerning macronutrient concentrations from different milk sampling protocols by investigating the accuracy of three milk sub-sampling protocols for estimating the true macronutrient concentrations of a complete feed. These sub-sampling protocols are (1) the mean of pre- and post-feed samples, (2) pre-feed samples only, and (3) post-feed samples only. In addition to the protocol comparison, the correlations between macronutrient concentrations and lactation characteristics are studied.

## Method

### Research Design

We designed a quantitative observational study comparing three sub-sampling protocols for macronutrient concentration estimation in human milk from a single pumping session, using foremilk, bulk milk, and/or hindmilk samples donated by healthy breastfeeding women. Additionally, the relation between macronutrient concentrations and lactation characteristics was analyzed. Ethical approval was granted by The Natural Sciences and Engineering Sciences Ethics Committee of the University of Twente (reference number 2021.118, 25 January 2022).

### Setting and Relevant Context

This study was conducted in the Netherlands, where 53% of newborns are exclusively breastfed at birth and 23% receive a combination of human milk and infant formula. Exclusive breastfeeding decreases to 49% after 2 weeks, 42% after 3 months, and 31% after 6 months (Bouwmeester & Staal, 2024). Breastfeeding rates are higher for full-term (*>* 37 weeks) pregnancies, amongst mothers with a higher education level, and mothers who are not born in the Netherlands (van Dommelen & Engelse, 2021). Mothers are entitled to at least 16 weeks of paid maternity leave, starting 4 to 6 weeks before the due date and lasting at least 10 weeks after childbirth, and are allowed to spend up to 25% of their work time breastfeeding or pumping during the first 9 months postpartum. Furthermore, breastfeeding support is a small aspect of the action program by the Dutch Ministry of Health, Welfare and Sport to support parents before, during, and after pregnancy (Ministerie van Volksgezondheid Welzijn en Sport, 2020).

### Sample

The target population consisted of healthy women who breastfed a single child with mature milk (at least 3 weeks postpartum). Exclusion criteria were non-singleton pregnancies, preterm born infants (< 37 weeks), serious lactation problems such as mastitis, and deviations from the milk collection protocol. Twenty women aged between 27 and 39 with a lactation period between 0.8 and 9 months postpartum participated in this study. Volunteers were recruited through midwife clinics in Enschede, the Netherlands, and the participant recruitment website of the University of Twente in Enschede. Participants in this study were experienced in using their own breast pump for regular pumping, due to work or personal reasons. One infant in this study received infant formula in addition to human milk, all infants older than 4.5 months received solid foods as well as human milk. This is in line with the Dutch guidelines to begin solid foods in addition to human milk when the infant is 4 to 6 months old (Stichting Voedingscentrum Nederland, 2024). Volunteers received no financial compensation for their participation in this study.

The final sample size of 15 is similar to the sample sizes of 20 and 13 participants, respectively, reported by Hytten (1954) and Woodward et al. (1989), although they followed some participants on multiple days. Despite the relatively small sample size, these data are a significant supplement to the minimal published data on changing macronutrient concentrations during one breastfeed.

### Measurement

**Figure 1.**
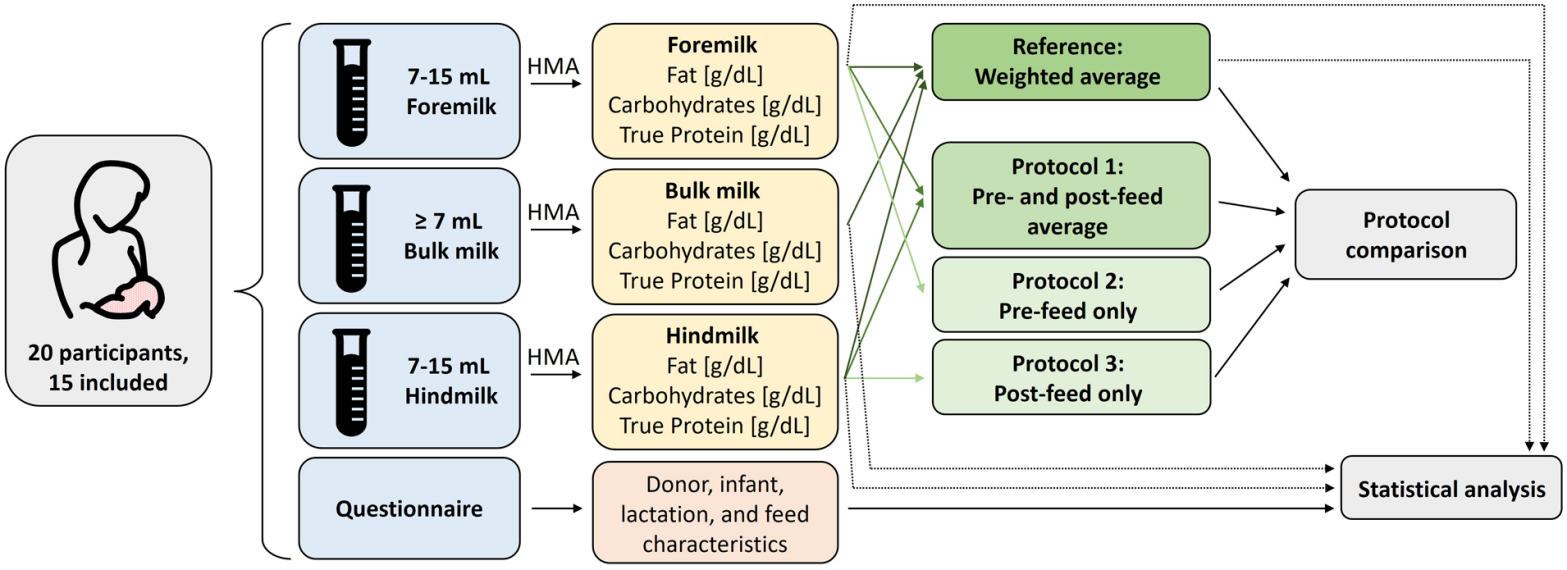
Schematic of the Research Design. *Note.* Twenty healthy lactating women donated foremilk, bulk milk, and hindmilk from a single breast for this study and filled in a questionnaire. Fifteen participants were included for analysis. Five participants were excluded due to a deviation from the sample collection protocol (deviating milk volumes) or measurement errors (0 protein concentration) in their milk samples. Milk samples were analyzed using a human milk analyzer (HMA, Miris, Sweden) for fat, true protein, and carbohydrate concentrations. The total feed composition from three sub-sampling protocols is compared to the volume-weighted average of the complete feed. Additionally, a statistical analysis of macronutrient concentrations and lactation characteristics was performed.

For this study, a sampling protocol was used where a complete feed from a single breast was pumped and collected in three parts: foremilk, hindmilk, and bulk milk (i.e., all milk between foremilk and hindmilk). The volume-weighted average of the macronutrient concentrations over the complete feed was used as a reference for the true macronutrient concentration. A schematic overview is shown in Figure 1. The volunteers completed a questionnaire to obtain information about donor age, gestation length, previous pregnancy history, infant birth sex (male/female), lactation period (infant age), average number of breastfeeds and/or pumping moments per day, time since previous feed, and additional food that the infant receives (none/vitamins/solids). The breast used for milk donation (left/right), total expressed milk volume, and any protocol deviations were recorded.

Three milk samples were collected per donor (see the Data Collection section). We measured fat, true protein, and carbohydrate concentrations for each milk sample with a human milk analyzer (HMA, Miris, Sweden) based on mid-infrared spectroscopy. Before the measurements, the frozen milk samples were thawed at room temperature and gently mixed. The Miris measurement protocol (Miris, 2022) was followed for heating, sonification, measurements, and cleaning. Each milk sample was measured only once, due to the limited volume of milk obtained with the sampling protocol. The manufacturer has reported that the HMA has an accuracy of 10% for fat and true protein, and 13% for carbohydrates (Miris, 2022). For hind milk samples, the expected fat concentration was above the recommended measurement range of the HMA (0.6 - 4 g/dl; Miris, 2022). Therefore, these samples were diluted 1:1 with deionized water before homogenization, and the macronutrient concentration results were corrected for this dilution step. The reliability of HMA results after dilution and correction was confirmed with additional measurements that showed equal macronutrient concentrations after dilution and correction compared to undiluted milk samples, within the error margins of the HMA.

### Data Collection

Milk samples were collected between April 2023 and October 2023. Milk was donated according to a step-by-step pumping protocol at home under the supervision of a female lactation researcher. Two donors followed the protocol without supervision. The volunteers used their own breast pump to extract milk from a single breast.

In this protocol, pumping was interrupted twice to collect separate samples of the foremilk, bulk milk, and hindmilk. Pumping was interrupted for the first time when 7–15 ml of milk was expressed. The expressed volume to this point was read from the bottle, and 7 ml of this foremilk was transferred with a syringe into a Falcon tube. Pumping was resumed, and milk was collected in the milk collection bottle together with any remaining foremilk. Pumping was interrupted a second time when the volunteer indicated that her breast was almost empty. The volume of the expressed milk was read from the bottle, and 7 ml of this bulk milk was transferred with a syringe into a second Falcon tube. Meanwhile, a new, empty bottle was connected to the pump, and pumping was resumed until the last 7–15 ml of milk was expressed. Again, the expressed volume was read from the bottle, and 7 ml of the hindmilk was transferred into a third Falcon tube. The donated milk samples were stored in the refrigerator overnight and frozen within 15 hours after donation (Donors 2 and 5), or frozen directly within 3 hours after donation (all other donors). The milk was stored at -20 ºC, and analyzed within 6 months after donation. The sample handling was equal for the three samples per donor to minimize potential effects of freezing time on the fat and protein concentrations (Kaya & Çınar, 2023).

The milk samples in this study were also used in our previous studies on the size distribution of human milk fat globules (Verveld et al., 2024) and the refractive index of human milk serum (de Wolf et al., 2024). All volunteers gave written informed consent before milk donation. We ensured secure storage and confidential treatment of all personal information and scientific data collected in this study.

### Data Analysis

The baseline characteristics from the questionnaire were tabulated per donor, and a summary containing averages and ranges (minimum-maximum) was obtained. The macronutrient concentrations and expressed milk volumes were tabulated per milk sample and visualized in graphs per macronutrient. Next, the true macronutrient concentration was calculated with a volume-weighted average of the macronutrient concentrations over the complete pumping session. This was used as the reference for comparison of the sub-sampling protocols. The volume-weighted average was calculated with the concentration [*c*] of a macronutrient (fat, carbohydrate, or true protein) and the volume *V* of each milk sample:

(1) $$\left[c_{weighted}\right]=\frac{\left[c_{foremilk}\right]*V_{foremilk}+\left[c_{bulkmilk}\right]*V_{bulkmilk}+\left[c_{hindmilk}\right]*V_{hindmilk}}{V_{foremilk}+V_{bulkmilk}+V_{hindmilk}}$$

Three milk sub-sampling protocols were analyzed:

1. Pre- and post-feed average: The macronutrient concentration was calculated with the unweighted mean of the concentrations in foremilk and hindmilk:

(2) $$\left[c_{pre-post}\right]=\frac{\left[c_{foremilk}\right]+\left[c_{hindmilk}\right]}{2}$$

2. Pre-feed only: For this protocol, the macronutrient concentration of foremilk [*c_foremilk_*] was used.
3. Post-feed only: For this protocol, the macronutrient concentration of hindmilk [*c_hindmilk_*] was used.

We compared the concentrations from Protocols 1, 2, and 3 to the volume-weighted average reference concentration using three statistical tests. First, we performed a linear regression analysis in Matlab (R2022a, The MathWorks Inc.) with functions *polyfit* and *polyval* to obtain the best linear fit (least-squares) and the standard error *δ* for each protocol and each macronutrient. The 95% confidence interval of the best fit was calculated by 2*δ*. Second, we used the function *corr* to calculate the Spearman rank correlation coefficient and *p*-value for each protocol and each macronutrient, compared to the reference. Spearman rank correlation was chosen instead of Pearson correlation, because the macronutrient concentrations were not necessarily normally distributed. Third, we used the function *signrank* for a two-sided Wilcoxon signed rank test, which is a nonparametric alternative to *t*-tests for dependent samples, to evaluate the presence of systematic biases that do not affect the correlation coefficient. Potential concentration biases were visualized in Bland-Altman plots, constructed per protocol and macronutrient.

A protocol was considered to be statistically different than the reference when either *p* > 0.05 for the Spearman rank correlation test (poor correlation) or *p* ≤ 0.05 for the Wilcoxon signed rank test (bias).

The correlations between macronutrient concentrations and non-binary questionnaire results were studied using the Spearman rank correlation. The Spearman rank correlation coefficient is calculated using the Matlab function *corr*. Four concentrations were studied per macronutrient: the foremilk concentration, bulk milk concentration, hindmilk concentration, and the volume-weighted average concentration. Additionally, the concentration increase was correlated with the questionnaire results, defined as the increase from foremilk to hindmilk as a percentage of the volume-weighted average concentration. Correlations with *p* ≤ 0.05 were considered to be statistically significant. For the binary variables of infant birth sex (male/female) and which breast was used for milk donation (left/right), the dataset was determined to be too small to obtain reliable comparisons between the groups.

## Results

### Sample Characteristics

Four donors were excluded from the analysis due to significant deviations in the sampling protocol: one donor could only express 1 ml of hindmilk after the second interruption, two donors expressed 20 ml of foremilk, and one donor expressed 35 ml hind milk. For one other donor, an unrealistic hindmilk protein concentration of 0 g/dl was measured. Since this is likely caused by an error in the dilution step for hind milk, these results were excluded from further analysis. The data from the remaining 15 participants were included for analysis. Table 1 contains a summary of the donor, infant, lactation, and milk donation characteristics of these 15 donors.

**Table 1.** Summary of the Donor, Infant, Lactation, and Donation Characteristics (*N* = 15).

Characteristics	Median (IQR)	*n* (%)
Donor age (years)	31 (3.8)	
Gestational length (weeks)	39.7 (2.1)	
Lactation period (weeks)	16.3 (15.0)	
Feedings per day (number)	8.0 (2.5)	
Time since previous feeding (hours)	3.0 (1.6)	
Time of day of milk donation (hh:mm)	10:40 (4:01)	
Total expressed milk volume (mL)	80 (37.0)	
Infant birth sex		
Male		8 (53%)
Female		7 (47%)
Breast for milk donation		
Left		8 (53%)
Right		7 (47%)

### Macronutrient Concentrations

**Figure 2.**
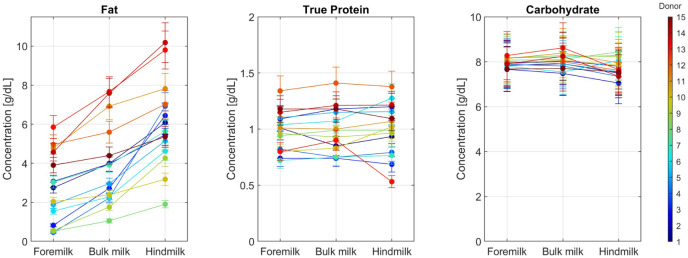
Human Milk Macronutrient Concentrations During a Pumping Session. *Note.* Fat, true protein, and carbohydrate concentrations are measured with a human milk analyzer (Miris) in the foremilk (first 7–15 ml), bulk milk (middle part), and hindmilk (last 7–15 ml) samples of 15 donors.

The macronutrient concentrations per milk sample are shown in Figure 2. For all donors, the fat concentration increased from foremilk (median 2.75 g/dl, min–max 0.46–5.85 g/dl) to bulk milk (median 3.91 g/dl, min–max 1.05–7.67 g/dl) to hindmilk (median 5.64 g/dl, min–max 1.90–10.18 g/dl). However, large differences exist between donors, as the fat content in the foremilk from some donors was higher than the fat content in the hindmilk from other donors. True protein concentrations also show wide variations between the donors (bulk median 0.99 g/dl, min–max 0.74 –1.41 g/dl), but were relatively constant throughout a feed. Carbohydrate concentrations show little variation between donors (bulk median 8.03 g/dl, min–max 7.48–8.62 g/dl) and throughout a feed.

### Sub-Sampling Protocol Comparison

**Figure 3.**
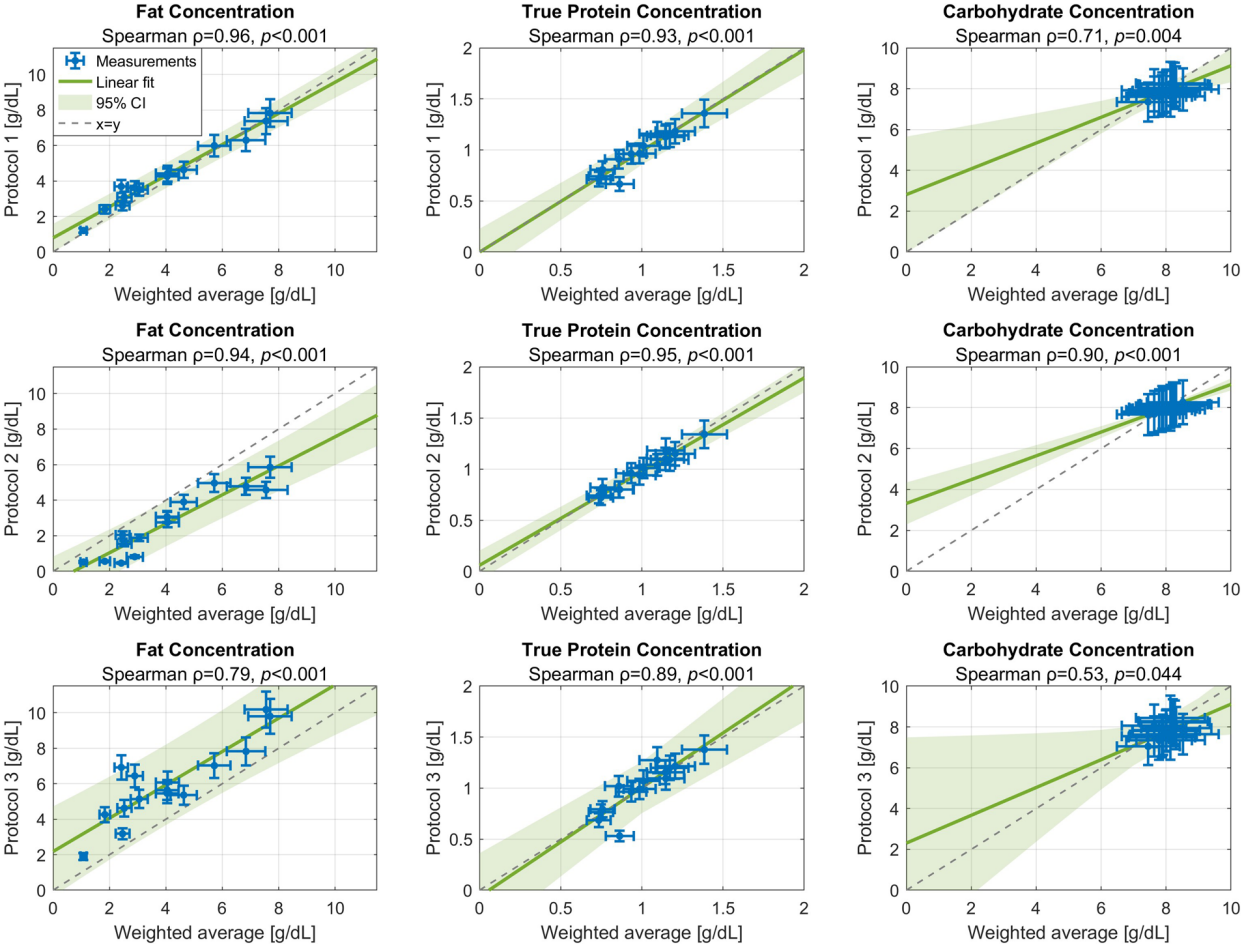
Measured Macronutrient Concentrations (Fat, True Protein, and Carbohydrates) for the Three Different Sub-Sampling Protocols, Versus the Volume-Weighted Average of a Complete Feed as Reference. *Note.* Protocol 1 corresponds to the unweighted average of foremilk and hindmilk (similar to pre- and post-feed sampling); Protocol 2 corresponds to foremilk only (similar to pre-feed sampling); and Protocol 3 corresponds to hindmilk only (similar to post-feed sampling). The Spearman rank correlation coefficient (ρ), *p*-value, and a linear regression fit (the green line) with a 95% confidence interval (CI; green area) on the protocol outcome versus reference are given for each protocol and macronutrient.

The macronutrient concentrations from Protocols 1, 2, and 3 were compared to the concentrations from the volume-weighted average in Figure 3. For both fat and true protein concentrations, strong correlations were found for all three sub-sampling protocols compared to the volume-weighted average (Spearman rank correlation coefficient ρ ≥ 0.79, *p* < 0.001). For carbohydrates, only a strong correlation was found for Protocol 2 (pre-feed only) compared to the volume-weighted average (ρ = 0.90, *p* < 0.001), while Protocols 1 (ρ = 0.71, *p* = 0.004) and 3 (ρ = 0.53, *p* = 0.044) were moderately correlated with the reference.

**Figure 4.**
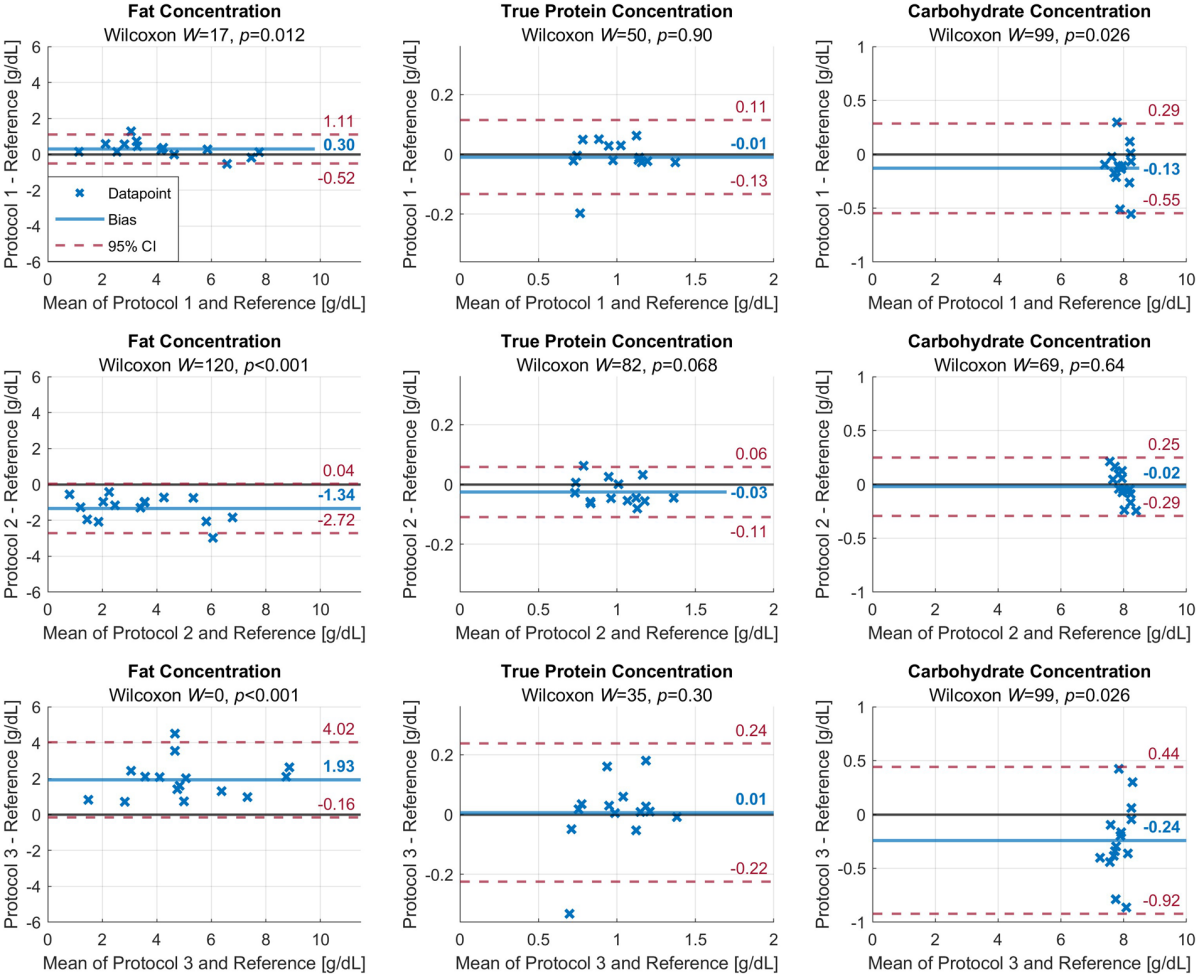
Bland-Altman Plots Comparing the Three Milk Sub-Sampling Protocols Per Macronutrient. *Note.* Protocol 1 is the unweighted average of foremilk and hindmilk (similar to pre- and post-feed sampling); Protocol 2 is foremilk only (similar to pre-feed sampling); and Protocol 3 is hindmilk only (similar to post-feed sampling). The volume-weighted average of a complete feed is used as a reference. The bias (blue) and the 95% confidence interval (CI; red) are given for each combination of protocols and macronutrient concentrations, as well as the positive signed-rank sum *W* and *p* value of the Wilcoxon signed rank test.

The Bland-Altman plots are shown in Figure 4. There is strong evidence that Protocols 2 and 3 do not result in an accurate estimate of the fat concentration, when compared to the volume-weighted average (Wilcoxon positive signed-rank sum *W* = 120 or *W* = 0, respectively, both *p* < 0.001). Due to the increase in fat content within one breastfeed, pre-feed milk only (Protocol 2) gives a significant underestimation of the fat concentration (-1.3 g/dl), while post-feed milk only (Protocol 3) overestimates the fat concentration of one complete feed (+1.9 g/dl). There is moderate evidence (*W* = 17, *p* = 0.012) that Protocol 1 creates a significant bias for estimating fat concentrations and that Protocols 1 and 3 create bias for estimating carbohydrate concentrations (*W* = 99, *p* = 0.026 for both protocols). The protein concentration is most constant throughout a breastfeed, and any sub-sampling protocol studied here shows good agreement with the volume-weighted average protein concentration.

### Questionnaire Correlation Analysis

The Spearman rank correlation coefficients were calculated between the macronutrient concentrations (foremilk concentration, bulk milk concentration, hindmilk concentration, volume-weighted average concentration, and concentration increase) and the variables from the questionnaire. Out of 114 combinations, 12 correlations were statistically significant with a *p* value < 0.05.

For the fat concentration increase during a pumping session, we found a strong positive correlation with the hours since the last feed (ρ = 0.85, *p* < 0.001), another strong positive correlation with the total volume of the expressed milk (ρ = 0.76, *p* < 0.001), and a moderately negative correlation with the average number of feedings per day (ρ = -0.57, *p* = 0.026). Additionally, we found moderately negative correlations between the fat concentration in foremilk and the hours since the last feed (ρ = -0.53, *p* = 0.041), as well as the fat concentration in foremilk and the volume of the expressed milk (ρ = -0.52, *p* = 0.048), but no significant correlation between the fat concentration in foremilk and the number of feedings per day (ρ = 0.23, *p* = 0.41). No statistically significant correlations were found in this dataset between the fat concentrations and the age of the mother, gestation length, lactation period (infant age), time of day of the milk donation, or the volume of the sub-sample.

For the protein concentrations, we found moderately to strong negative correlations with the lactation period (foremilk: ρ = -0.54, *p* = 0.038; bulk milk: ρ = -0.54, *p* = 0.036; hind milk: ρ = -0.72, *p* = 0.003; weighted average: ρ = -0.58, *p* = 0.023; increase during a feed: ρ = -0.57, *p* = 0.026). Lastly, we found moderately positive correlations for the average number of feeds per day with the protein concentration in hindmilk (ρ = 0.58, *p* = 0.024) and the protein concentration increase during a feed (ρ = 0.56, *p* = 0.030). No statistically significant correlations were found between the protein concentrations and the age of the mother, gestation length, hours since the last feed, volume of the expressed milk, time of day of the milk donation, or the volume of the sub-sample. For the carbohydrate concentrations, no statistically significant correlations were found with any of the feed, donor, or lactation characteristics.

## Discussion

In this study, we aimed to investigate the accuracy of three milk sub-sampling protocols for estimating the true macronutrient concentrations of a complete feed. The strength of this study is that the volumes per sub-sample were known, which allowed us to calculate a volume-weighted average over the complete feed and use this parameter as a reference. In this way, different sub-sampling protocols could be compared precisely, giving a unique insight into potential biases within and between protocols.

### Applicability of Sub-Sampling Protocols

A good sub-sampling protocol should result in macronutrient concentrations that have a strong correlation with the volume-weighted average concentrations, but without a significant systematic bias. Based on the correlations and significance limit of *p <* 0.05, all three sub-sampling protocols have this strong correlation for all three studied macronutrient concentrations. However, all three sub-sampling protocols resulted in a significant bias for the fat concentration (*p <* 0.05).

The fat concentration bias of +0.30 g/dl from Protocol 1 was observed to be smaller than the measurement error in the case of high-fat milk samples. This implies that Protocol 1 could be applied for milk samples with fat concentrations above 3.0 g/dl measured with an HMA due to the HMA error of 10% for fat (Miris, 2022). This limit value is dependent on the accuracy of the measurement method. For milk samples with a lower fat concentration in the complete feed, the use of Protocol 1 gives a systematic overestimation of the fat concentration. The error that we obtained between the pre- and post-feed average fat concentration compared to the volume-weighted average is most often larger than the 5% error as reported by Prentice et al. (1981) on the data of Hytten (1954). The use of Protocol 2 (pre-feed only) and Protocol 3 (post-feed only) for measuring fat concentrations is strongly discouraged, due to the significant under- and overestimation by these protocols.

The biases in carbohydrate concentration from Protocol 1 (-0.13 g/dl) and Protocol 3 (-0.24 g/dl) were also statistically significant (*p <* 0.05), but they are much smaller than the measurement error of ∼ 1 g/dl of the HMA for carbohydrates (based on a 13% error on an average carbohydrate concentration of ∼8 g/dl). Therefore, either protocol could be used to measure carbohydrate concentrations. For true protein concentrations, any of the three sub-sampling protocols could be used, as no significant biases were found.

The final impact of any bias in the estimation of the fat concentration will differ per study that applies a sub-sampling protocol. A systematic bias is more impactful when absolute caloric intakes are calculated than when relative differences in milk from the same participant between different time points are studied. Supported by the outcomes of this study, researchers in the field of human lactation can now base their choice of a milk sampling strategy not only on practical or ethical arguments, but also on the accuracy that we determined for the various sub-sampling protocols.

It should be noted that Hytten (1954) sampled a breastfeed in sub-volumes of 12 ml, while we used a volume of 7–15 ml for fore- and hindmilk. In more recent literature, pre- and post-feed samples of only 1–2 ml were used (Suwaydi, Lai, Rea, et al., 2023). The influence of the milk sample volume on the sub-sampling protocol accuracy could not be investigated with the current dataset, but is of interest for future research, because the fat concentration is expected to change also within the first and last milliliters. Due to the minimum volume of 3 ml milk that is necessary for measurements with the Miris HMA, the use of creamatocrit methods with a smaller sample volume and higher accuracy (Tie et al., 2021) would be more suitable than a HMA for a future study.

### Lactation Characteristics

In addition to the protocol comparison, the correlations between macronutrient concentrations and lactation characteristics were studied. We found that three lactation characteristics (including hours since the last feed, the volume of a feed, and the average number of feedings per day) were significantly correlated to the fat increase during a pumping session. Additional Spearman rank tests showed that these lactation characteristics were also moderately correlated with each other (*p <* 0.05 for each combination). The most significant correlation was found between the fat increase during a pumping session and the hours since the last feed. This could be partially attributed to the moderate correlation between the fat concentration in foremilk and the time since the previous feed, where the fat concentration in foremilk was observed to be generally lower for milk expressions after a longer time interval since the previous feed. A similar trend was also observed by Daly et al. (1993), who explained the larger milk fat concentration increase during a feed by a larger degree of breast emptying. In general, the presence of lactation characteristics that influence the fat increase during a pumping session emphasizes the need for milk sampling protocols that are valid under all lactation circumstances.

Lastly, the decrease in protein concentration over the lactation period (age of infant) that we found was already mentioned in our previous work based on all donated mature bulk milk samples from this dataset (de Wolf et al., 2024). Here, we would like to add that the same relation was also observed by Saarela et al. (2005). For both protein and carbohydrate concentrations in general, our dataset is also consistent with earlier research that showed constant concentrations throughout a feed (Saarela et al., 2005). Therefore, only one milk sample taken at any point in a breastfeed is sufficient to represent the protein and carbohydrate concentrations of the complete feed.

### Limitations

This study was limited by the relatively small number of donors. All participants lived in one region of the Netherlands and delivered term infants (37–42 weeks). Maternal body mass index (BMI), type of delivery, and diet were not recorded. We could not rule out the influences of socioeconomic conditions, diet, BMI, and type of delivery on the accuracy of sub-sampling protocols, as there is no substantial dataset yet that describes how these factors influence the change in macronutrient concentrations throughout a feed or pumping session. Similarly, the correlations between the macronutrient concentrations and lactation characteristics that were found for these 15 participants with term infants cannot be generalized to all cultures or lactation circumstances based on this study alone, but the agreement with other literature indicates that most aspects can be considered universal. Another limitation of this dataset is that most milk samples were donated in the morning. Therefore, we did not have enough data points to study circadian rhythms and their potential effect on the accuracy of sub-sampling protocols.

The inclusion criteria on the volumes of fore- and hindmilk assured a well-bounded study, but also caused a high exclusion rate (~20%) and prohibited investigation of the influence of the milk sample volume on the sub-sampling protocol accuracy. Altering the milk sample volume or inclusion criteria could affect the reported bias values in a meaningful way. In contrast, it is unlikely that the overall conclusions of this study would change significantly by including more milk samples according to our current criteria. Nevertheless, we recommend increasing the sample size and acquiring replicates instead of single measurements in future studies to reduce random errors and improve study quality.

## Conclusions

In this study, all three sub-sampling protocols showed strong correlations with the reference fat concentration. However, neither sub-sampling protocol was accurate, because all three sub-sampling protocols had a significant bias from the reference. The mean of pre- and post-feed samples (Protocol 1), based on fore- and hindmilk volumes of 7–15 ml, gave a bias in the fat concentration of +0.30 g/dl and could therefore be accurate enough for high-fat milk samples where this bias is smaller than the measurement error from the human milk analyzer. Pre-feed samples only (Protocol 2) and post-feed samples only (Protocol 3) should not be used to estimate the fat concentration of a complete feed, due to their large bias of -1.3 g/dl and +1.9 g/dl, respectively. For the protein and carbohydrate concentrations, only one milk sample taken at any moment during a breastfeed is sufficient to accurately represent the protein and carbohydrate concentrations of the complete feed. It is recommended that future research focuses on a detailed model that describes fat content during a feed or pumping session with higher temporal resolution.
